# Supplementary material for: Magnetic bead-based separation of pneumococcal serotypes
Source: Cell Rep Methods. 2023 Feb 21;3(2):100410. doi: 10.1016/j.crmeth.2023.100410 (PMC10014298; doi:10.1016/j.crmeth.2023.100410)
Supplement: Document S1. Figure S1 and Tables S1–S3 [file mmc1.pdf]

Cell Reports Methods, Volume *3*

Supplemental information

Magnetic bead-based separation of pneumococcal serotypes

Anna York, Emily Huynh, Sidiya Mbodj, Devyn Yolda-Carr, Maikel S. Hislop, Haley Echlin, Jason W. Rosch, Daniel M. Weinberger, and Anne L. Wyllie

Supplemental Information

Table S1. KingFisher Flex MBS Protocol. Related to STAR Methods.

**Pick up tips**
96DW tip comb
**Collect Beads**
Count 2 seconds 5
**Mix**
Release Beads<br>Medium mix 0:01:00min<br>Count 3 seconds 1
**Release Beads**
Fast 00:00:05
**Leave**
Leave Plate

Table S2. Serotypes and corresponding antisera pools used for MBS (rabbit antiserum; SSI Diagnostica, Hillerød, Denmark) and for serotyping (ImmuLex™ Pneumotest; SSI Diagnostica).Related to STAR Methods.

**Serotype**	**Pooled Antisera for Neufeld**	**ImmuLex™ Pneumococcus Antisera**
12F	Pool E #16733<br>Pool R #16741	Pool E #52394<br>Pool R #52401
23F	Pool H #16736<br>Pool Q #16740	Pool H #52397<br>Pool Q #52400
3	Pool B #16728<br>Pool R #16741	Pool B #52391<br>Pool R #52401
14	Pool P #16739<br>Pool H #16736	Pool P #52399<br>Pool H #52397
19A	Pool B #16728<br>Pool P #16739	Pool B #52391<br>Pool P #52399
15A/B/C	Pool H #16736<br>Pool S #16742	Pool H #52397<br>Pool S #52402
2 (D39)	Pool A #16725<br>Pool T #16743	Pool A #52390<br>Pool T #52403
35B	Pool G #16735	Pool G #52396
11A/B	Pool D #16731<br>Pool T #16743	Pool D #52393<br>Pool T #52403

Table S3. KingFisher Flex DNA extraction protocol. Related to STAR Methods.

**Pick up tips**
96DW tip comb
**Predigest**
56ºC<br>Preheat on<br>Mixing medium 0:10:00 minutes
**Heat kill pk 95 ºC**
Preheat off<br>Mixing medium 0:10:00 minutes
**Initial Mixing**
Release bead 0:00:00<br>Slow mix 0:01:00min<br>Medium mix 0:04:00min<br>Loop1
**Add Proteinase K and Binding**
Add 10 µl pro k, 25 µl binding beads and 530 µl binding buffer
**PK Digest**
Release bead 0:00:00<br>Preheat on 65ºC<br>Mix Fast 0:15:00 min<br>Collect beads 5 count 1 second<br>Collect beads: Count 5 time 0
**Wash 1**
Release beads 20 second bottom mix<br>Mixing 0:00:10 bottom mix<br>0:00:10 Fast mix<br>Loop 3 times<br>Collect beads: Count 5
**Wash 2**
Release beads<br>0:00:20 FAST<br>Mixing 0:00:10 bottom mix<br>0:00:10 Fast mix<br>Loop 2 times<br>Collect beads: 4 count 1 second
**Wash 3**
Release beads<br>0:00:00<br>Mix<br>0:00:30 Fast<br>Collect beads: Count 5 seconds 0
**Dry**
0:02:00 Dry type (outside well)
**Elution 1**
Release Beads<br>0:00:00<br>Heating and Cooling<br>Preheat ON<br>75°C<br>Mixing 0:00:15 bottom mix<br>0:00:45 Medium mix<br>Loop 6 times<br>Collect Beads<br>Count 1 seconds 0

**Final Beads**
Mixing<br>0:02:00 slow
**Elution 2**
Release Beads<br>0:00:00<br>Heating and Cooling<br>Preheat ON<br>75°C<br>Mixing 0:00:15 bottom mix<br>0:00:45 Medium mix<br>Loop 6 times<br>Collect Beads<br>Count 1 seconds 0
**Final Beads**
Mixing<br>0:02:00 slow
**Leave Tip**
Tip Plate
**HOLD Temp**
Elution Plate 1<br>10°C

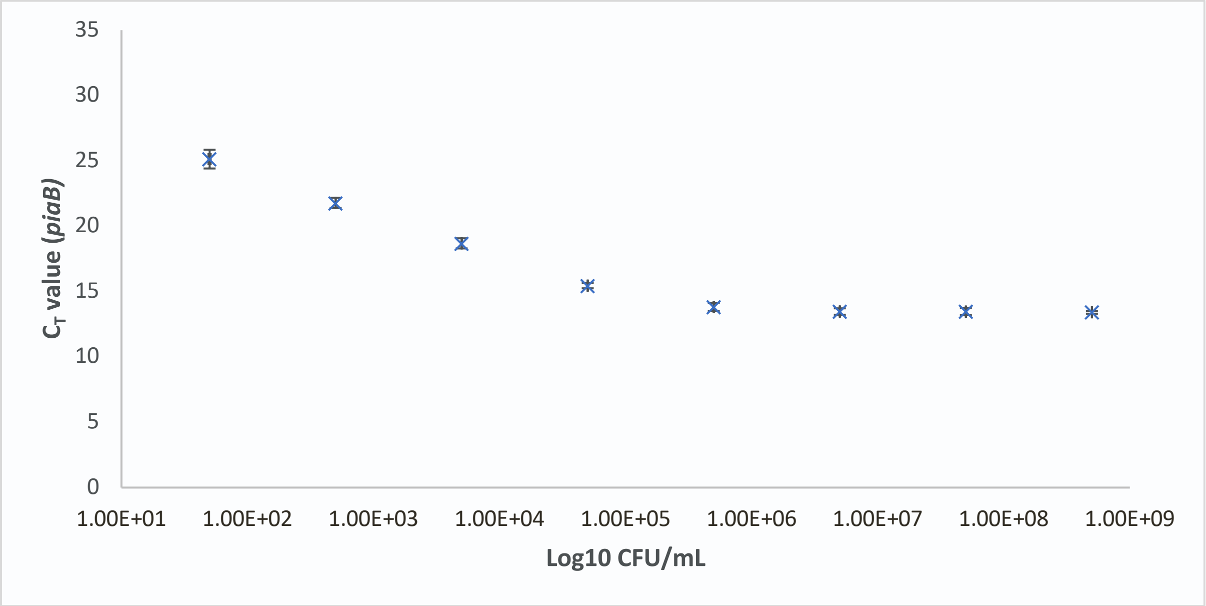

Figure S1. Detection of pneumococcus gene piaB (C_T value) when culture-enriched saliva samples were tested with qPCR, and the corresponding CFU/mL of S. pneumoniae 19A that was spiked into each raw saliva sample. Raw saliva was confirmed to be pneumococcus-negative (C_T >40) by qPCR towards piaB. Data shown as mean and standard deviation of biological triplicate data. Related to STAR Methods.
