# Supplementary material for: Magnetic bead-based separation of pneumococcal serotypes
Source: Cell Rep Methods. 2023 Feb 21;3(2):100410. doi: 10.1016/j.crmeth.2023.100410 (PMC10014298; doi:10.1016/j.crmeth.2023.100410)
Supplement: Document S2. Article plus supplemental information [file mmc5.pdf]

Cell Reports Methods

Article

Magnetic bead-based separation of pneumococcal serotypes

Graphical abstract

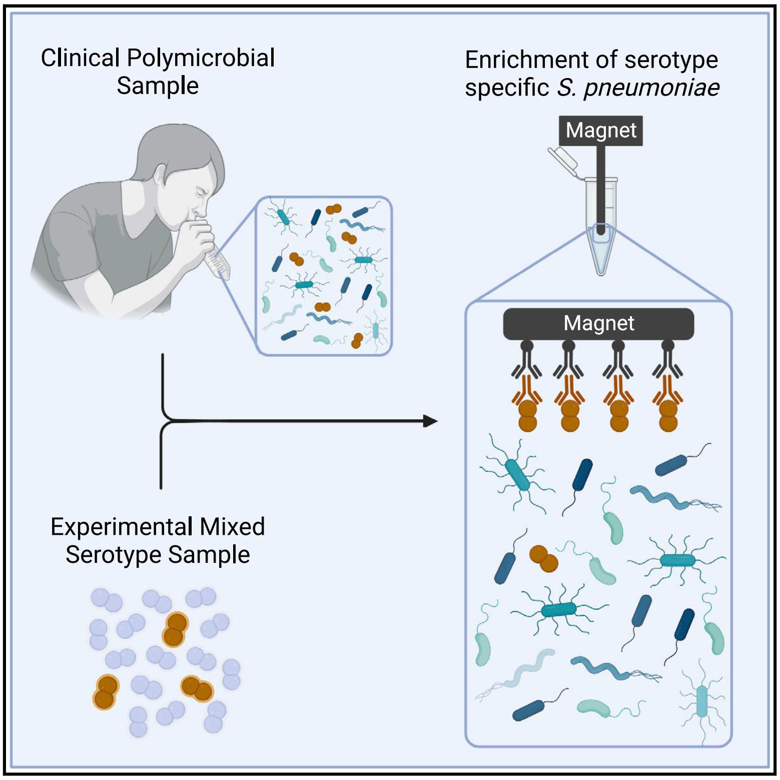

Highlights

- MBS technique achieves 100- to 900-fold enrichment of *S. pneumoniae* from mixed samples
- Known pneumococcal serotypes can be enriched from clinical saliva samples
- The MBS technique shows improvement over traditional plating and isolating techniques

Authors

Anna York, Emily Huynh, Sidiya Mbodj, ..., Jason W. Rosch, Daniel M. Weinberger, Anne L. Wyllie

Correspondence

anna.york@yale.edu

In brief

Streptococcus pneumoniae is carried asymptomatically in healthy individuals but causes disease in vulnerable populations. York et al. describe a magnetic bead-based separation technique that can enrich for *S. pneumoniae* from a polymicrobial clinical sample (saliva) or from a mixed-serotype experimental sample, facilitating studies aimed at improving vaccines and treatments.

York et al., 2023, Cell Reports Methods *3*, 100410
February 27, 2023 © 2023 The Authors.
https://doi.org/10.1016/j.crmeth.2023.100410

Cell Reports Methods

CellPress
OPEN ACCESS

Article

Magnetic bead-based separation of pneumococcal serotypes

Anna York,[1,4,*] Emily Huynh,[1] Sidiya Mbodj,[1] Devyn Yolda-Carr,[1] Maikel S. Hislop,[1,2] Haley Echlin,[3] Jason W. Rosch,[3] Daniel M. Weinberger,[1] and Anne L. Wyllie[1]

[1]Department of Epidemiology of Microbial Diseases, Yale School of Public Health, New Haven, CT 06510, USA
[2]Department of Life Sciences and Chemistry, Utrecht University of Applied Sciences, 3584 CS Utrecht, the Netherlands
[3]Department of Infectious Disease, St. Jude Children's Research Hospital, Memphis, TN 38105, USA
[4]Lead contact
*Correspondence: anna.york@yale.edu
https://doi.org/10.1016/j.crmeth.2023.100410

MOTIVATION Use of molecular methods has improved identification of pneumococci in saliva samples; however, isolating pneumococcus, particularly from mixed/complex samples, can be challenging. We developed a magnetic bead-based separation method to enrich samples for pneumococci in order to make isolation easier and less labor intensive.

SUMMARY

The separation of pneumococcal serotypes from a complex polymicrobial mixture may be required for different applications. For instance, a minority strain could be present at a low frequency in a clinical sample, making it difficult to identify and isolate by traditional culture-based methods. We therefore developed an assay to separate mixed pneumococcal samples using serotype-specific antiserum and a magnetic bead-based separation method. Using qPCR and colony counting methods, we first show that serotypes (12F, 23F, 3, 14, 19A, and 15A) present at ~0.1% of a dual serotype mixture can be enriched to between 10% and 90% of the final sample. We demonstrate two applications for this method: extraction of known pneumococcal serotypes from saliva samples and efficient purification of capsule switch variants from experimental transformation experiments. This method may have further laboratory or clinical applications when the selection of specific serotypes is required.

INTRODUCTION

Streptococcus pneumoniae (pneumococcus) is an opportunistic pathogen that resides asymptomatically in the upper respiratory tract of many healthy adults and children worldwide. This asymptomatic colonization is a pre-requisite for the development of pneumococcal disease, including upper respiratory tract infections (such as otitis media), lower respiratory tract infections (such as pneumonia), and invasive pneumococcal disease (IPD) (such as meningitis and bacteremia). Pneumococcal disease often occurs in the very young, elderly, or immunocompromised.[1] Pneumococcus is a leading cause of lower respiratory disease, and in 2016 alone, it contributed to more deaths than all other etiologies combined.[2]

The capsular polysaccharide (CPS) is the outermost layer of encapsulated strains of *S. pneumoniae*, and more than 100 antigenically distinct serotypes have been identified.[3] Pneumococcal conjugate vaccines (PCVs) are highly effective against pneumococcal disease but only cover up to 20 of these serotypes. While pneumococcal disease declined following the introduction of PCVs, a concomitant increase in disease caused by non-vaccine serotypes occurred. This emergence of non-vaccine serotypes in carriage and invasive disease is called serotype replacement.[4] Serotype replacement occurs for two reasons: first the opening of a new niche in which existing strains expressing capsules not targeted by the vaccine can thrive. Second, vaccine-targeted strains can acquire the capsule biosynthesis cassette from a different serotype, allowing them to evade vaccine-induced immunity. Serotype switching occurs when the *cps* locus from one *S. pneumoniae* serotype (or related species) is transferred into the genetic backbone of another *S. pneumoniae* serotype by transformation.[5] Genetic exchange between two *S. pneumoniae* serotypes requires co-colonization of two or more serotypes.

In addition to naturally occurring serotype switches,[6–8] researchers have been generating *cps* switch mutants in the lab for nearly 100 years. The first capsule switch experiments conducted by Griffith in 1928 were accomplished by mixing avirulent, unencapsulated pneumococci with virulent, but killed, encapsulated strains and injecting this mixture into a mouse.

© 2023 The Authors.
This is an open access article under the CC BY license (http://creativecommons.org/licenses/by/4.0/).

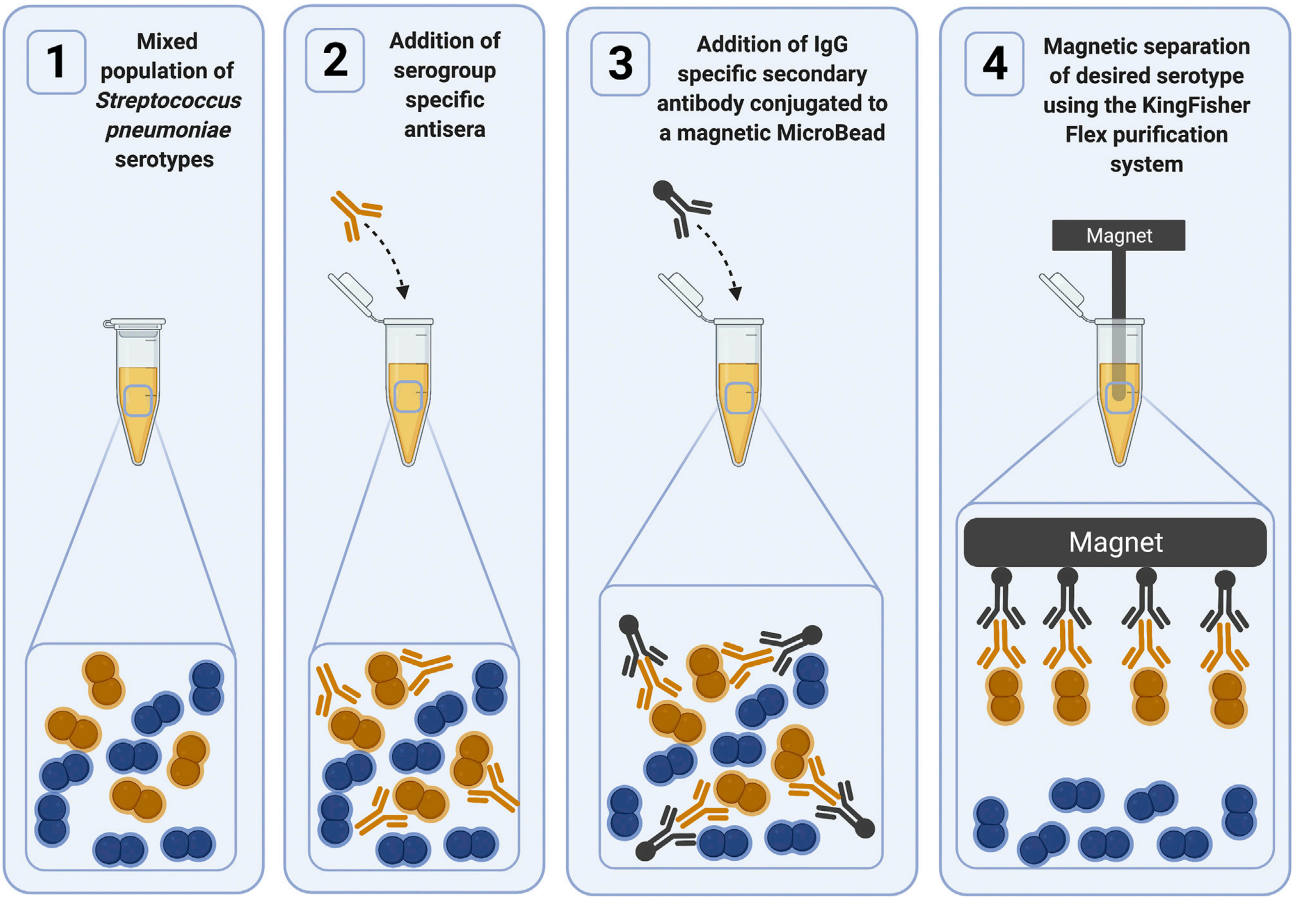

Figure 1. An overview of the magnetic bead-based separation (MBS) method
To a dual serotype mixture (1), antisera specific for the desired serotype are added. (2) Following brief wash steps, IgG-specific secondary antibody conjugated to a magnetic bead is incubated (3), and finally, the desired cells are extracted using the KingFisher Flex Purification System (4).
Created with BioRender.com.

The capsule-switched strains could then be isolated from the mouse.[9] More recently, generating *cps* switch mutants in the lab has been accomplished using various genetic cassettes.[10,11] These types of studies have permitted the generation of a number of capsule switch mutants, and this allows for detailed experimental evaluation of the relative importance of capsule and genetic background for different phenotypes.[8,12–16] Current methods for generating capsule-switched variants require the use of selectable markers, are labor intensive, and are not easily scalable. Methods that allow for separation of multiple serotypes could allow for higher throughput generation of capsule switch mutants and could be used alongside qPCR or sequencing to investigate capsule switching in a competitive manner (i.e., in the presence of multiple DNA donors or recipients).

There is also a need to isolate individual pneumococcal strains from clinical samples. Nasopharyngeal swabs have long been considered the gold standard sample type for the detection of carriage of *S. pneumoniae*,[17] but recent studies have demonstrated utility for saliva to improve the detection of carriage in adults.[14,15,18] Although testing saliva improves the detection of pneumococci when using molecular methods (such as qPCR), it can be challenging for the isolation of live pneumococcal colonies due to the density and diversity of bacteria present in saliva. A method that enables the separation of pneumococci, in a serotype-specific manner, from other species present in saliva would be useful for clinical and laboratory studies alike.

We developed a magnetic bead-based separation (MBS) method that requires no selection markers and can be used to extract live pneumococci, of a known serotype, from a mixture of pneumococci or from clinical samples containing other bacteria (such as saliva).

RESULTS

The MBS method allows for enrichment of serotype-specific *S. pneumoniae* from a mixed or polymicrobial sample (such as saliva). MBS uses a primary antibody (either antisera or monoclonal antibody) directed toward the serotype or serogroup of interest; a commercially available secondary antibody conjugated to a magnetic bead is then added. The magnet in the KingFisher Flex Purification System is used to remove the antibody and its associated serotype of interest from the remaining mixture

CellPress
OPEN ACCESS

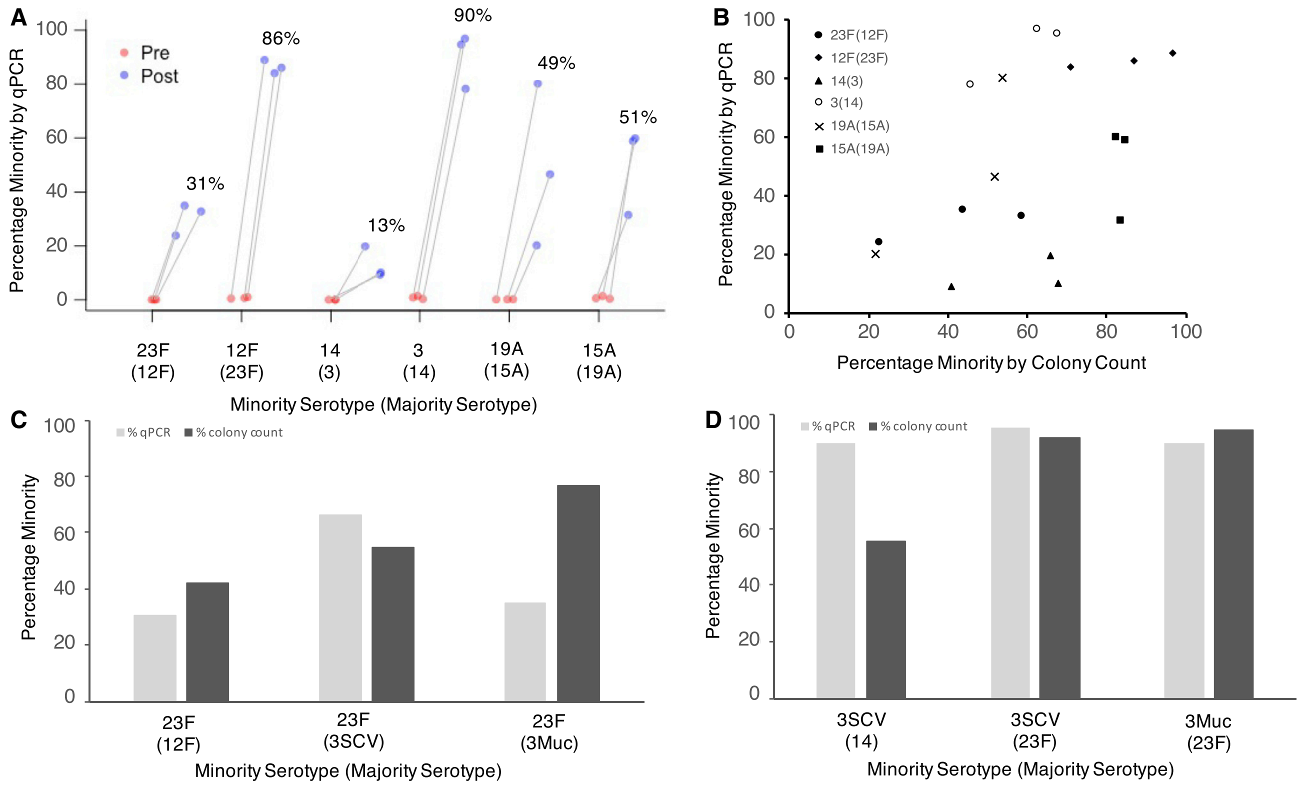

Figure 2. Efficiency of minority strain enrichment using the MBS method

(A) Percentage minority serotype present prior to MBS (Pre) and after MBS (Post) for six serotypes in three initial serotype pairs. The average percentage minority in the post sample is presented above the data points. Minority and majority serotypes are displayed on the x axis in the following format: minority (majority). Triplicate results are shown for each of three serotype pairs where each serotype of the pair was tested as the minority serotype.

(B) Comparison of percentage minority serotype present after MBS (Post) as determined by qPCR and colony counting methods.

(C) Averages of triplicate data are shown for 23F (when with a majority of 12F), and averages of duplicate data were plotted for 23F (when with a majority of 3SCV and 3Muc). Minority and majority serotypes are displayed on the x axis in the following format: minority (majority).

(D) Averages of triplicate data are shown for 3SCV (when with a majority of 14), and averages of duplicate data were plotted for 3SCV and 3Muc (when with a majority of 23F). Percentage minority from both colony counting and qPCR methods is shown. Minority and majority serotypes are displayed on the x axis in the following format: minority (majority).

See also Tables S4 and S5.

(Figure 1). The enriched sample is plated on blood agar plates and incubated overnight at 37°C with 5% CO_2. Bacterial colonies of interest can then be isolated from the blood agar plate.

The MBS proof-of-concept experiments showed that for all six serotypes, the minority serotype was successfully enriched from ~0.1% starting percentage to between 13% (serotype 14) and 90% (serotype 3) after MBS, corresponding to a 100- to 900-fold enrichment (Figure 2A). The final percentage of the minority varied between serotypes but was relatively consistent between the three replicates. There was generally good concordance in the estimated MBS efficiency as determined by the qPCR and colony counting (Figure 2B); however, efficiency determined by colony counting seemed to be higher and lower than with qPCR for serotype 14 and 3, respectively. Eight colonies from each elution plate were selected at random, and in every single case, minority serotype colonies were identified by serotyping (Table 1). This demonstrates that this technique can be used to recover a desired serotype from a dual mixture.

A secondary analysis was conducted to identify whether serotype 3Muc was also enriched with a similar efficiency as serotype 3SCV and to gain insight into how separation efficiency varies when the majority serotype of the pair is altered. MBS was conducted on Pair 4 (23F and 3SCV) and Pair 5 (23F and 3Muc). The results were compared with MBS results obtained previously for enrichment of minority serotypes 23F or 3SCV when paired with another majority serotype (namely serotype 12F and serotype 14 from Pair 1 and Pair 2, respectively). The percentage enrichment for both 23F and 3 remained similar even when the majority serotype of the pair was altered (Figures 2C and 2D). Furthermore, it demonstrates that the MBS method permits successful enrichment of both small non-mucoid colony variant (SCV) and mucoid variants of serotype 3, and that the efficiency is similar regardless of the morphology. In all cases minority serotype single colonies were isolated from the elution plate by selection of single colonies and confirmed to be the desired serotype using SSI latex agglutination (Table 1).

Table 1. Total number of colonies on the plain blood agar elution plate (out of eight selected at random) that were positive for the minority serotype (as determined by SSI latex agglutination) following MBS

Minority serotype in serotype pair	Majority serotype in serotype pair	Number of minority serotype colonies (Rep1)	Number of minority serotype colonies (Rep2)	Number of minority serotype colonies (Rep3)
23F	12F	5	5	5
12F	23F	8	6	5
14	3 (SCV)	4	4	3
3 (SCV)	14	7	6	6
19A	15A	1	7	3
15A	19A	7	6	5
23F	3(SCV)	4	2	N/A
3(SCV)	23F	6	8	N/A
23F	3(Muc)	6	8	N/A
3(Muc)	23F	5	7	N/A

Positive results reported were those that tested positive with minority serotype antisera and negative with majority serotype antisera.

The primary analysis specifically used serotype pairs that could be distinguished using two unique pools of antisera. MBS was then tested on eight serotype pairs using only a single antisera pool. A total of six antisera pools (H, P, B, E, R, H, and Q) were tested, and all were able to successfully enrich an ~0.1% minority serotype to between 10% and 99% in the final sample (Figure 3A).

Additional analysis aimed to determine whether enrichment was constant at different percentage minorities. The 23F and 12F pair were used with the majority serotype (12F) remaining constant at 1×10^7 CFU and the minority serotype (23F) at four different concentrations in the initial sample. Enrichment of the minority serotype can be achieved even when the starting percentage of a minority serotype is as low as 1×10^3 CFU. However, as the initial percentage minority decreases, the percentage minority recovered following MBS also decreases. For initial samples containing 5×10^4, 1×10^4, 5×10^3, and 1×10^3 CFUs of minority serotype 23F, the corresponding percentages of 23F present in the final samples were 27%, 14%, 8%, and 6% respectively, as determined by qPCR, or 49%, 23%, 13%, and 9%, respectively, as determined by colony counting (Figure 3B).

In order to separate serogroups that share reactivity to one antiserum pool, the MBS method should be used with only a single antiserum pool. We therefore investigated outcomes when using one or two antisera pools and compared the efficiency of antisera pools in the presence of different majority serotypes. MBS of serotype 14 from a majority serotype 3, using both antisera Pool H and Pool P, resulted in the final sample containing ~13% of serotype 14. However, use of only Pool H or Pool P, at an equal final volume to the combined pools, resulted in serotype 14 being 10% and 45% of the final samples, respectively. Therefore, in this example, Pool P alone achieves the greatest efficiency of MBS, but in the absence of knowing which antisera is more efficient, and if the serotype pairs permit dual use, it would be prudent to combine both antisera pools. Furthermore, we confirm that the overall efficiency of enrichment achieved by any antisera pool is not only dependent upon the minority serotype alone, but also the majority serotype. The final percentage of serotype 14 following MBS (using Pool P) from a majority serotype 23F is 99%, more than double the percentage of serotype 14 present following MBS (using Pool P) from a majority serotype 3.

Generation of capsule switch mutants by transformation

Transformation reactions were conducted individually, to establish the success of each individual transformation using the standard methods (in the absence of the MBS method). D39S-Δ*cps*:SweetJanus was incubated with genomic DNA (gDNA) from encapsulated D39 (serotype 2) as a positive control. For the positive control, 8/8 colonies selected were confirmed to be serotype 2 (indicating successful transformation). D39S-Δ*cps*:SweetJanus was also successfully transformed with gDNA from serotype 23F and serotype 35B, with 7/8 and 8/8 colonies selected confirmed to be 23F and 35B, respectively. Conversely, 0/8 colonies selected were confirmed to be 12F or 11B, suggesting that transformation may not have occurred or may have occurred at very low efficiency for these gDNA donors.

Mixed transformation reactions (i.e., gDNA from four serotypes combined with one recipient strain) show that even in the absence of cell separation, it is possible to isolate transformants for 23F and 35B, with 9/32 and 11/32 confirmed to be these serotypes, respectively. Similarly, to the results seen in the individual transformations, transformants of 12F or 11B were not identified (0/32) from the mixed transformation in the absence of MBS. The mixed transformations that were subsequently processed using the MBS method to enrich for the desired serotype showed that 23F, 35B, and 11B were successfully transformed, with 8/8, 5/8, and 7/8 colonies identified to be 23F, 35B, and 11B, respectively (Table 2). This confirms that 11B is able to transform into D39SΔ*cps*:SweetJanus at the *cps* locus, but this likely occurs at a lower efficiency, making it challenging to isolate without using MBS. For serotype 12F, colonies were observed on the blood agar plate (BAP) following MBS, however 0/8 were identified to be 12F transformants, therefore this transformation may only occur at very low frequencies, under very specific conditions, or not at all. Of the eight colonies selected from the cell separation enriching for 12F, 6/8 were serotype 23F, 1/8 were serotype 11B, and only 1/8 was untransformed. For serotype

CellPress
OPEN ACCESS

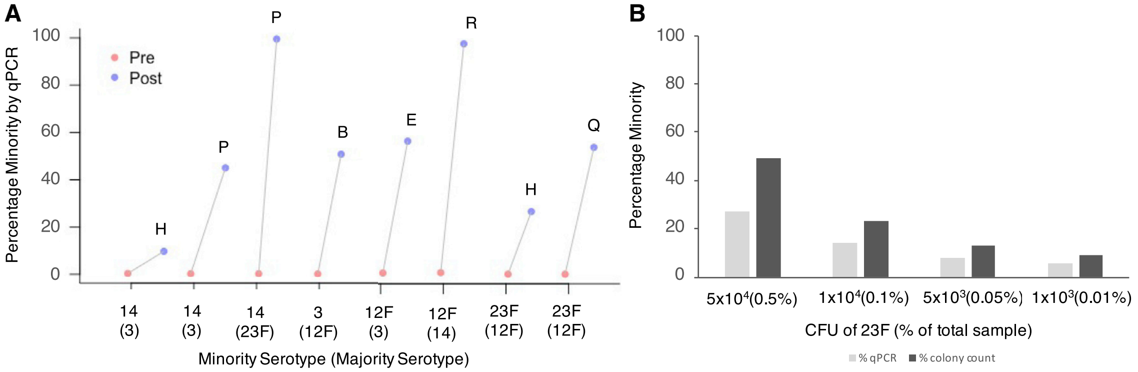

Figure 3. Efficiency of minority enrichment using the MBS method on eight serotypes

(A) Percentage minority serotype present prior to MBS (Pre) and after MBS (Post).

(B) Percentage minority 23F present after MBS (Post) as determined by qPCR and colony counting. Minority and majority serotypes are displayed in the following format: minority (majority). Results shown are singlicate data points only.

See also Tables S4 and S6.

35B, 1/3 was serotype 23F, and 2/3 were untransformed. For serotype 11B, 1/8 was serotype 23F. The presence of these contaminating serotypes suggests that the antisera/antibodies used in MBS have some non-specific cross-reactivity, and the presence of contaminating 23F in all samples suggests that it may be particularly "sticky."

Enriching for 19A from saliva

To determine if the MBS method could be used to enrich for a known serotype in pneumococcus-positive saliva, we spiked two saliva samples (A and B) which tested qPCR-negative for pneumococcal genes *piaB* and *lytA*, with varying concentrations of serotype 19A, and we compared the success of identifying pneumococcal colonies in the presence and absence of MBS (Table 3). For both saliva A and saliva B, at all concentrations of 19A, the MBS method resulted in equal or improved isolation of pneumococcal colonies. In saliva A, the MBS method was still able to enrich for pneumococcus when the concentration of 19A was 5×10^1 CFU/mL in raw saliva; however for Saliva B, the MBS method was only successful at a 19A concentration of 5×10^3 CFU/mL in raw saliva. The sensitivity of this assay is therefore dependent upon not only the concentration of pneumococci in the sample but also the composition of saliva itself, and it may vary from sample to sample. The MBS method was then tested on six clinical saliva samples that were qPCR-positive for *piaB* and for which the serotype was already known (Table 4). Each sample had varying success with isolation of pneumococcus using the standard culture-based dilution method,[19] and the MBS method enriched for the known serotype making isolation of colonies easier. Selected colonies were optochin tested, and serotypes were confirmed using the SSI latex agglutination assay.

Table 2. Number of positive transformations for each serotype from a mixed transformation containing gDNA of 12F, 23F, 35B, and 11B, in the presence of cell separation (8 colonies picked per sample) and in the absence of cell separation picked (total of 32 colonies picked)

Serotype	Control (no cell separation) number of transformants	Cell separation number of transformants
12F	0/32	0/8
23F	9/32	8/8
35B	11/32	5/8
11B	0/32	7/8

DISCUSSION

We developed the MBS method that can enrich for a desired serotype from a mixed-serotype sample in a laboratory setting. Enrichment using the MBS method was demonstrated for six serotypes (23F, 12F, 3, 14, 15A, and 19A), including two serotypes with more unique capsules (serotype 3 and serotype 14). We were able to demonstrate two use cases for this method: separation of capsule switch mutants (from mixed transformation experiments) and enrichment of pneumococcus from saliva samples.

In the primary analysis used to develop the MBS method, we show that all six of the minority serotypes investigated (23F, 12F, 14, 3, 19A, and 15A) can be successfully enriched from ~0.1% of an initial mixed-serotype sample to up between 13% and 90% in the final sample. The inclusion of serotype 3 (which exists as small colony and mucoid variants) and serotype 14 (which has an uncharged capsule)[20,21] in this panel showed that this method is suitable for serotypes with rarer capsule properties. Two methods—colony counting and qPCR—were employed in order to assess efficiency of the MBS method. The estimates from both methods were broadly concurrent, but there are a few examples where the efficiency estimates do differ. This may be explained by the formation of varying chain lengths in pneumococcus, so if the two serotypes in a pair form vastly different length chains, the estimations of efficiency may be biased. A serotype that readily forms chains would result in an underestimation of its presence in the sample using the colony counting method, but qPCR would provide a more accurate estimation. Despite some differences in efficiency estimates

Table 3. Isolation of optochin-sensitive pneumococcal colonies (number of optochin-sensitive/number of colonies selected), using the standard dilution method compared with the MBS method

	Number of 19A colonies identified in Saliva A		Number of 19A colonies identified in Saliva B	
CFU/mL of *S. pneumoniae* 19A	Saliva A with MBS method	Saliva B with standard dilution method	Saliva A with MBS method	Saliva B with standard dilution method
5×10^4	15/16	15/16	15/16	1/16
5×10^3	15/16	5/16	5/16	0/16
5×10^2	16/16	2/16	0/16	0/16
5×10^1	6/24	0/24	0/24	0/24

Two pneumococcal-negative saliva samples (A and B) were spiked with four concentrations of serotype 19A. Pure pneumococcal colonies were identified by a zone of inhibition around the optochin disk, and any colonies that were mixed colonies (i.e., those with a zone of inhibition but some secondary growth [a non-pneumococcal contaminant] growing within the zone of inhibition or had satellite colonies appearing withing the zone of inhibition) were considered to be successful isolation of pneumococcus.

between colony counting and qPCR methods, we were able to successfully isolate minority serotype colonies after MBS in all cases. This demonstrates a tangible utility for this method in the laboratory setting. When separating a mixture of cells, only a small number of colonies must be isolated to identify the desired serotype. This method therefore allows for the easy recovery of serotype-specific *S. pnuemoniae* isolates.

In the secondary supporting analysis, we compared how enrichment of a minority serotype varied when in the presence of different majority serotypes. A minority serotype 23F was paired with a majority serotype of either 12F or 3, and minority serotype 3 was paired with a majority serotype of either 14 or 23F. With minority 23F, some variation in efficiency of MBS was noted when the majority serotype was changed, however for minority serotype 3, the enrichment efficiency remained very similar despite the change in majority serotype pair. This suggests that the serotype with which the minority is mixed may have some impact on the efficiency of MBS, but it is likely primarily determined by the avidity of the antisera for the desired serotype. Unlike the majority of pneumococcal serotypes, serotype 3 utilizes the synthase-dependent pathway for CPS production, resulting in non-covalently bound CPS that can be released from the glycolipids or synthase.[21] The CPS of serotype 3 is not covalently linked to the peptidoglycan and can be released,[22] which leads to a reduction in the protective effect of anti-type 3 CPS antibodies induced by the PCV13,[23] we were therefore surprised to find that the MBS method can successfully extract serotype 3 from a mixed sample. This success may be explained by the fact that the cells are not actively growing and likely therefore not releasing CPS into the environment. Furthermore, it is intriguing but reassuring that the efficiency of enrichment between mucoid and SCV serotype 3 is very similar; the MBS method can be successfully used on serotype 3 samples, which are of particular interest due to the reduced effectiveness of PCV13 on serotype 3 IPD.[24–26]

We demonstrate that good separation can be achieved with only one unique antiserum, meaning that serotypes with cross-reactivity to one antiserum can still be separated using this method. As expected, we demonstrate that the efficiency of enrichment achieved by each of the two antisera pools is not equal, and therefore, depending on the desired serotype, one antisera may be preferred over another. Furthermore, enrichment of a serotype can occur even when a serotype is present at only 0.01% of the total sample (1×10^3 minority serotype with 1×10^7 majority serotype).

Having optimized the MBS method, we evaluated its potential for laboratory applications. The MBS method allows for competitive transformation experiments with multiple donor serotypes in a single mixed reaction. This may be beneficial for investigation of the impact of transformation of non-cps loci in capsule switching. After initial selection for transformants on selection media, the MBS method can be used to separate out the individual transformants in a serotype-specific manner. Mixed transformations would permit higher throughput generation of capsule-swapped variants, the potential to determine comparative efficiency, and a significant reduction in BAP usage and labor intensity. However, in the absence of MBS, while isolation of different serotypes is comparable to that observed in individual transformations, the benefits are offset by the lengthy and time-consuming process of serotype screening each isolate by latex agglutination. Therefore, to harness the true benefit of mixed transformations, a simple and easy technique to select for different serotypes is required. The MBS method was used to isolate multiple serotypes from a mixed sample of four serotypes. The MBS method outperformed the individual transformations and the mixed transformation (without MBS) by successfully isolating an additional serotype (11B), which was not isolated using the other methods. This suggests that the MBS method may be particularly useful to enrich for serotypes which transform with low efficiency. The MBS technique was not 100% specific, and a small amount of cross-reactivity was observed; however, because each sample is enriched for the desired serotype, and the serotype of each colony is confirmed by latex agglutination, these contaminants are of little concern for this particular application.

We also show that the MBS method can be modified to successfully enrich for pneumococci from saliva, which is highly polymicrobial. While not investigated in this study, we expect that the MBS method will also perform well on other polymicrobial samples such as oropharyngeal swabs. In this study, we isolated pneumococcus of a known serotype from spiked-saliva samples (19A) and then subsequently showed the method to be successful in enriching pneumococcus in

CellPress
OPEN ACCESS

Table 4. Isolation of optochin-sensitive pneumococcal colonies (number of optochin-sensitive/number of colonies selected), from each of six clinical saliva samples using the standard dilution method compared with the MBS method

Saliva sample number (Serotype)	Number of optochin-sensitive colonies identified with MBS method	Number of optochin-sensitive colonies identified with standard dilution method
1 (15B)	29/32	0/32
2 (23F)	4/13	1/8
3 (15B)	8/8	1/8
4 (19A)	7/8	4/8
5 (11A)	8/8	3/8
6 (3)[a]	7/8	2/8

[a]Sample 6 (serotype 3) used a modified protocol (SSI antisera only). Pure pneumococcal colonies were identified by a zone of inhibition around the optochin disk (STAR Methods).

six clinical saliva samples known to be positive for serotypes 15B/C, 23F, 11A, 19A, and 3. Enrichment is possible even in saliva samples where pneumococci is present at very low concentrations (5×10^1 CFU/mL), for which isolation of pneumococci using standard methods is typically very challenging. This permits easy identification and isolation of pneumococci present in saliva at concentrations too low to detect using standard dilution and plating methods. The use of SSI antisera alone on a polymicrobial sample such as saliva was problematic due to antisera reactivity with non-pneumococcal bacteria present in saliva. In general, we found that the SSI antisera outperformed mAbs in terms of total number of pneumococcal colonies isolated, and we hypothesize that this is due to the increased avidity of antisera (presence of IgA, IgM) that agglutinates pneumococci, increasing the overall yield during MBS. Therefore, to take advantage of the increased avidity of antisera and simultaneously the high specificity of mAbs, we combined both in the primary incubation step, but we only targeted the mAb in the secondary antibody step. This method was found to be superior for the enrichment of serotypes 15B/C, 23F, 11A, and 19A from saliva; however, we found that enrichment of serotype 3 did not occur with the combined use of SSI and mAb. Instead, for serotype 3, SSI antisera alone resulted in enrichment (7/8 colonies), while combined use of SSI antisera and mAb performed worse (0/8 colonies) than the standard dilution and plating method (2/8 colonies). It is possible that this complication occurs due to the release of the serotype 3 capsule from the pneumococcus,[23] and the mAb (but not the antisera) is readily sequestered by the unbound capsule, thereby hindering enrichment. Following MBS from all saliva samples, the elution was not 100% pure pneumococci, however, contaminating non-pneumococcal bacteria were reduced, and identification and selection of single pneumococcal colonies were improved when compared with the standard dilution and plating method. The enrichment observed varies depending on concentration of pneumococci present in the sample, but also on the saliva composition itself. The composition of bacterial community in saliva varies between different age groups,[27] and so the success of the MBS method will likely vary accordingly; however in this study, we show the MBS method working well on clinical saliva samples from children, adults, and the elderly. Since the MBS method can work on saliva containing very low concentrations of pneumococci, it may be particularly useful for the isolation of minority serotypes in samples obtained from multiply colonized individuals. Previous research shows that 52% of Dutch primary school children tested positive for multiple pneumococcal serotypes,[28] however, conventional serotyping methods often result in an underestimation of multiply colonized individuals.[29] Detection of multiple serotypes is possible using serologic, biochemical (mass spectroscopy and nuclear magnetic resonance), and genotypic (sequencing, qPCR, and microarrays) methods. However, until now, attempting to isolate minority serotypes by conventional methods (single colony selection) has been laborious and time consuming.[21]

In conclusion, the MBS method allows for the successful enrichment of a minority serotype from a dual sample containing two *S. pneumoniae* serotypes belonging to different serogroups. Using this method, an initial sample containing 0.01%–0.1% of a desired serotype can be enriched to up to 90% in the final sample. Enrichment to between 10% and 90% was demonstrated for six minority serotypes, and half of the commercially available antisera pools (Pools B, E, H, P, Q, R, and S) were tested. We demonstrate two different applications for this technique: separating capsule switch variants from mixed transformation experiments and enriching for pneumococci of a known serotype from saliva. The MBS technique can be used successfully to enrich for serotypes which are present at very low levels in both mixed cultures and more complex polymicrobial sample types (such as saliva), making it a versatile and important technique for a multitude of applications.

Limitations of the study

A key limitation of the MBS method, in general, is that due to cross-reactivity within serogroups, SSI antisera pools can only be used to separate *S. pneumoniae* serotypes belonging to different serogroups. To circumvent this limitation, serotype-specific mAbs can be used, as shown in the clinical saliva experiments. In addition to this, the use of SSI Omni serum to target all or multiple pneumococcal serotypes would only work for some applications; for example, this may be appropriate for separating encapsulated and non-encapsulated pneumococci, but it would perform poorly in saliva due to cross-reactivity with

non-pneumococcal streptococci present in this sample type. Another limitation is the total proportion of minority cells that can be recovered. While enrichment from 0.1% up to >10% has been demonstrated, it is worth noting that only a small proportion (~1%) of the total minority cells present in the initial mixture are successfully extracted. This may be overcome by increasing antibody incubation periods or antibody concentration to ncrease binding capacity.

STAR★METHODS

Detailed methods are provided in the online version of this paper and include the following:

- KEY RESOURCES TABLE
- RESOURCE AVAILABILITY
  - Lead contact
  - Materials availability
  - Data and code availability
- EXPERIMENTAL MODEL AND SUBJECT DETAILS
  - Microbe strains
  - Human subjects
- METHOD DETAILS
  - Magnetic bead-based separation (MBS) method
  - Proof of concept and primary analysis
  - Secondary analyses
  - Colony counting to quantify separation efficiency
  - Sample R equation
  - Sample S equation
  - Serotyping of colonies to confirm separation efficiency
  - Real-time qPCR to confirm separation efficiency
  - Demonstrating applications for the MBS method
  - Isolating pneumococci from a saliva sample
- QUANTIFICATION AND STATISTICAL ANALYSIS

SUPPLEMENTAL INFORMATION

Supplemental information can be found online at https://doi.org/10.1016/j.crmeth.2023.100410.

ACKNOWLEDGMENTS

This work was supported by R01AI123208 from NIAID/NIH (D.M.W.). The content is solely the responsibility of the authors and does not necessarily represent the official views of the National Institutes of Health.

AUTHOR CONTRIBUTIONS

Conceptualization, D.M.W. and A.L.W.; methodology, A.Y., D.M.W., and A.L.W.; investigation, A.Y., E.H., S.M., M.S.H., D.Y.-C., H.E., and J.W.R.; resources, D.M.W. and A.Y.; data curation, A.Y. and D.M.W.; writing – original draft, A.Y.; writing – review & editing, D.M.W., A.L.W., and D.Y.-C.; visualization, A.Y. and D.M.W.; supervision, A.L.W., D.M.W., and J.W.R.; project administration, A.Y.; funding acquisition, D.M.W. and A.L.W.

DECLARATION OF INTERESTS

D.M.W. has received consulting fees from Pfizer, Merck, GSK, Affinivax, and Matrivax and is PI on research grants from Pfizer and Merck to Yale. A.L.W. has received consulting fees from Pfizer and is PI on research grants from Pfizer to Yale.

INCLUSION AND DIVERSITY

We support inclusive, diverse, and equitable conduct of research.

Received: May 2, 2022
Revised: November 18, 2022
Accepted: January 26, 2023
Published: February 21, 2023

REFERENCES

1. Centers for Disease Control and Prevention CDC; Advisory Committee on Immunization Practices (2010). Updated recommendations for prevention of invasive pneumococcal disease among adults using the 23-valent pneumococcal polysaccharide vaccine (PPSV23). MMWR Morb. Mortal. Wkly. Rep. *59*, 1102–1106.
2. GBD 2016 Lower Respiratory Infections Collaborators; Blacker, B., Khalil, I.A., Rao, P.C., Cao, J., Zimsen, S.R.M., Albertson, S.B., Deshpande, A., Farag, T., Abebe, Z., et al. (2018). Estimates of the global, regional, and national morbidity, mortality, and aetiologies of lower respiratory infections in 195 countries, 1990–2016: a systematic analysis for the Global Burden of Disease Study 2016. Lancet Infect. Dis. *18*, 1191–1210. https://doi.org/10.1016/S1473-3099(18)30310-4.
3. Ganaie, F., Saad, J.S., McGee, L., van Tonder, A.J., Bentley, S.D., Lo, S.W., Gladstone, R.A., Turner, P., Keenan, J.D., Breiman, R.F., and Nahm, M.H. (2020). A new pneumococcal capsule type, 10D, is the 100th serotype and has a large *cps* fragment from an oral Streptococcus. mBio *11*, e00937–e1020. https://doi.org/10.1128/mBio.00937-20.
4. Weinberger, D.M., Malley, R., and Lipsitch, M. (2011). Serotype replacement in disease after pneumococcal vaccination. Lancet *378*, 1962–1973. https://doi.org/10.1016/S0140-6736(10)62225-8.
5. Nahm, M.H., Brissac, T., Kilian, M., Vlach, J., Orihuela, C.J., Saad, J.S., and Ganaie, F. (2019). Pneumococci can become virulent by acquiring a new capsule from oral streptococci. J. Infect. Dis. *222*, 372–380. https://doi.org/10.1093/infdis/jiz456.
6. Chochua, S., Metcalf, B.J., Li, Z., Walker, H., Tran, T., McGee, L., and Beall, B. (2017). Invasive serotype 35B pneumococci including an expanding serotype switch lineage, United States, 2015–2016. Emerg. Infect. Dis. *23*, 922–930. https://doi.org/10.3201/eid2306.170071.
7. Moore, M.R., Gertz, R.E., Jr., Woodbury, R.L., Barkocy-Gallagher, G.A., Schaffner, W., Lexau, C., Gershman, K., Reingold, A., Farley, M., Harrison, L.H., et al. (2008). Population snapshot of emergent Streptococcus pneumoniae serotype 19A in the United States, 2005. J. Infect. Dis. *197*, 1016–1027. https://doi.org/10.1086/528996.
8. Croucher, N.J., Kagedan, L., Thompson, C.M., Parkhill, J., Bentley, S.D., Finkelstein, J.A., Lipsitch, M., and Hanage, W.P. (2015). Selective and genetic constraints on pneumococcal serotype switching. PLoS Genet. *11*, e1005095. https://doi.org/10.1371/journal.pgen.1005095.
9. Griffith, F. (1928). The significance of pneumococcal types. J. Hyg. *27*, 113–159.
10. Li, Y., Thompson, C.M., and Lipsitch, M. (2014). A modified janus cassette (Sweet Janus) to improve allelic replacement efficiency by high-stringency negative selection in Streptococcus pneumoniae. PLoS One *9*, e100510. https://doi.org/10.1371/journal.pone.0100510.
11. Echlin, H., and Rosch, J.W. (2020). Advancing genetic tools in Streptococcus pneumoniae. Genes *11*, 965. https://doi.org/10.3390/genes11090965.
12. Kelly, T., Dillard, J.P., and Yother, J. (1994). Effect of genetic switching of capsular type on virulence of Streptococcus pneumoniae. Infect. Immun. *62*, 1813–1819.
13. Hathaway, L.J., Brugger, S.D., Morand, B., Bangert, M., Rotzetter, J.U., Hauser, C., Graber, W.A., Gore, S., Kadioglu, A., and Mühlemann, K. (2012). Capsule type of Streptococcus pneumoniae determines growth phenotype. PLoS Pathog. *8*, e1002574. https://doi.org/10.1371/journal.ppat.1002574.

CellPress
OPEN ACCESS

14. Abruzzo, A.R., Aggarwal, S.D., Sharp, M.E., Bee, G.C.W., and Weiser, J.N. (2022). Serotype-dependent effects on the dynamics of pneumococcal colonization and implications for transmission. mBio *13*, e00158–e00222. https://doi.org/10.1128/mbio.00158-22.

15. An, H., Qian, C., Huang, Y., Li, J., Tian, X., Feng, J., Hu, J., Fang, Y., Jiao, F., Zeng, Y., et al. (2022). Functional vulnerability of liver macrophages to capsules defines virulence of blood-borne bacteria. J. Exp. Med. *219*, e20212032. https://doi.org/10.1084/jem.20212032.

16. Trzciński, K., Li, Y., Weinberger, D.M., Thompson, C.M., Cordy, D., Bessolo, A., Malley, R., and Lipsitch, M. (2015). Effect of serotype on pneumococcal competition in a mouse colonization model. mBio *6*, e00902–e00915. https://doi.org/10.1128/mBio.00902-15.

17. Satzke, C., Turner, P., Virolainen-Julkunen, A., Adrian, P.V., Antonio, M., Hare, K.M., Henao-Restrepo, A.M., Leach, A.J., Klugman, K.P., Porter, B.D., et al. (2013). Standard method for detecting upper respiratory carriage of Streptococcus pneumoniae: updated recommendations from the World Health Organization pneumococcal carriage working group. Vaccine *32*, 165–179. https://doi.org/10.1016/j.vaccine.2013.08.062.

18. Krone, C.L., Wyllie, A.L., van Beek, J., Rots, N.Y., Oja, A.E., Chu, M.L.J.N., Bruin, J.P., Bogaert, D., Sanders, E.A.M., and Trzciński, K. (2015). Carriage of Streptococcus pneumoniae in aged adults with influenza-like-illness. PLoS One *10*, e0119875. https://doi.org/10.1371/journal.pone.0119875.

19. Trzciński, K., Bogaert, D., Wyllie, A., Chu, M.L.J.N., van der Ende, A., Bruin, J.P., van den Dobbelsteen, G., Veenhoven, R.H., and Sanders, E.A.M. (2013). Superiority of trans-oral over trans-nasal sampling in detecting Streptococcus pneumoniae colonization in adults. PLoS One *8*, e60520. https://doi.org/10.1371/journal.pone.0060520.

20. Kamerling, J.P. (1999). Pneumococcal polysaccharides: a chemical view. In In Streptococcus pneumoniae: molecular biology and mechanisms of disease, pp. 81–114.

21. Geno, K.A., Gilbert, G.L., Song, J.Y., Skovsted, I.C., Klugman, K.P., Jones, C., Konradsen, H.B., and Nahm, M.H. (2015). Pneumococcal capsules and their types: past, present, and future. Clin. Microbiol. Rev. *28*, 871–899. https://doi.org/10.1128/CMR.00024-15.

22. Cartee, R.T., Forsee, W.T., Schutzbach, J.S., and Yother, J. (2000). Mechanism of type 3 capsular polysaccharide synthesis inStreptococcus pneumoniae. J. Biol. Chem. *275*, 3907–3914. https://doi.org/10.1074/jbc.275.6.3907.

23. Choi, E.H., Zhang, F., Lu, Y.-J., and Malley, R. (2016). Capsular polysaccharide (CPS) release by serotype 3 pneumococcal strains reduces the protective effect of anti-type 3 CPS antibodies. Clin. Vaccine Immunol. *23*, 162–167. https://doi.org/10.1128/CVI.00591-15.

24. Horácio, A.N., Silva-Costa, C., Lopes, J.P., Ramirez, M., Melo-Cristino, J., Portuguese Group for the Study of Streptococcal Infections; Vaz, T., Gião, M., Ferreira, R., Fonseca, A.B., et al. (2016). Serotype 3 remains the leading cause of invasive pneumococcal disease in adults in Portugal (2012–2014) despite continued reductions in other 13-valent conjugate vaccine serotypes. Front. Microbiol. *7*, 1616. https://doi.org/10.3389/fmicb.2016.01616.

25. Silva-Costa, C., Brito, M.J., Pinho, M.D., Friães, A., Aguiar, S.I., Ramirez, M., and Melo-Cristino, J.Portuguese Group for the Study of Streptococcal Infections; Portuguese Study Group of Invasive Pneumococcal Disease of the Pediatric Infectious Disease Society (2018). Pediatric complicated pneumonia caused by Streptococcus pneumoniae serotype 3 in 13-valent pneumococcal conjugate vaccinees, Portugal, 2010–2015. Emerg. Infect. Dis. *24*, 1307–1314. https://doi.org/10.3201/eid2407.180029.

26. Slotved, H.-C., Dalby, T., Harboe, Z.B., Valentiner-Branth, P., Casadevante, V.F.d., Espenhain, L., Fuursted, K., and Konradsen, H.B. (2016). The incidence of invasive pneumococcal serotype 3 disease in the Danish population is not reduced by PCV-13 vaccination. Heliyon *2*, e00198. https://doi.org/10.1016/j.heliyon.2016.e00198.

27. Xu, X., He, J., Xue, J., Wang, Y., Li, K., Zhang, K., Guo, Q., Liu, X., Zhou, Y., Cheng, L., et al. (2015). Oral cavity contains distinct niches with dynamic microbial communities. Environ. Microbiol. *17*, 699–710. https://doi.org/10.1111/1462-2920.12502.

28. Wyllie, A.L., Chu, M.L.J.N., Schellens, M.H.B., van Engelsdorp Gastelaars, J., Jansen, M.D., van der Ende, A., Bogaert, D., Sanders, E.A.M., and Trzciński, K. (2014). Streptococcus pneumoniae in saliva of Dutch primary school children. PLoS One *9*, e102045. https://doi.org/10.1371/journal.pone.0102045.

29. Huebner, R.E., Dagan, R., Porath, N., Wasas, A.D., Klugman, K.P., and Klugman, K.P. (2000). Lack of utility of serotyping multiple colonies for detection of simultaneous nasopharyngeal carriage of different pneumococcal serotypes. Pediatr. Infect. Dis. J. *19*, 1017–1020.

30. Tóthpál, A., Kardos, S., Laub, K., Nagy, K., Tirczka, T., van der Linden, M., and Dobay, O. (2015). Radical serotype rearrangement of carried pneumococci in the first 3 years after intensive vaccination started in Hungary. Eur. J. Pediatr. *174*, 373–381. https://doi.org/10.1007/s00431-014-2408-1.

31. Wyllie, A.L., Fournier, J., Casanovas-Massana, A., Campbell, M., Tokuyama, M., Vijayakumar, P., Warren, J.L., Geng, B., Muenker, M.C., Moore, A.J., et al. (2020). Saliva or nasopharyngeal swab specimens for detection of SARS-CoV-2. N. Engl. J. Med. *383*, 1283–1286. https://doi.org/10.1056/NEJMc2016359.

32. Rayack, E.J., Askari, H.M., Zirinsky, E., Lapidus, S., Sheikha, H., Peno, C., Kazemi, Y., Yolda-Carr, D., Liu, C., Grubaugh, N.D., et al. (2022). Routine saliva testing for SARS-CoV-2 in children: partnering with childcare centers in the greater new haven community. Preprint at medRxiv. https://doi.org/10.1101/2022.05.05.22274434.

33. Wyllie, A.L., Mbodj, S., Thammavongsa, D.A., Hislop, M.S., Yolda-Carr, D., Waghela, P., Nakahata, M., Watkins, A.E., Vega, N.J., York, A., et al. (2022). Persistence of pneumococcal carriage among older adults in the community despite COVID-19 mitigation measures. Preprint at medRxiv. https://doi.org/10.1101/2022.06.28.22276654.

34. Allegrucci, M., and Sauer, K. (2007). Characterization of colony morphology variants isolated from Streptococcus pneumoniae biofilms. J. Bacteriol. *189*, 2030–2038. https://doi.org/10.1128/JB.01369-06.

35. Pimenta, F.C., Roundtree, A., Soysal, A., Bakir, M., du Plessis, M., Wolter, N., von Gottberg, A., McGee, L., Carvalho, M.d.G., and Beall, B. (2013). Sequential triplex real-time PCR assay for detecting 21 pneumococcal capsular serotypes that account for a high global disease burden. J. Clin. Microbiol. *51*, 647–652.

36. Wyllie, A.L., Rümke, L.W., Arp, K., Bosch, A.A.T.M., Bruin, J.P., Rots, N.Y., Wijmenga-Monsuur, A.J., Sanders, E.A.M., and Trzciński, K. (2016). Molecular surveillance on Streptococcus pneumoniae carriage in non-elderly adults; little evidence for pneumococcal circulation independent from the reservoir in children. Sci. Rep. *6*, 34888. https://doi.org/10.1038/srep34888.

STAR★METHODS

KEY RESOURCES TABLE

REAGENT or RESOURCE	SOURCE	IDENTIFIER
**Antibodies**		
Pooled Antisera for Neufeld (Pool A)	SSI Diagnostica	#16725
Pooled Antisera for Neufeld (Pool B)	SSI Diagnostica	#16728
Pooled Antisera for Neufeld (Pool D)	SSI Diagnostica	#16731
Pooled Antisera for Neufeld (Pool E)	SSI Diagnostica	#16733
Pooled Antisera for Neufeld (Pool G)	SSI Diagnostica	#16735
Pooled Antisera for Neufeld (Pool H)	SSI Diagnostica	#16736
Pooled Antisera for Neufeld (Pool P)	SSI Diagnostica	#16739
Pooled Antisera for Neufeld (Pool Q)	SSI Diagnostica	#16740
Pooled Antisera for Neufeld (Pool R)	SSI Diagnostica	#16741
Pooled Antisera for Neufeld (Pool S)	SSI Diagnostica	#16742
Pooled Antisera for Neufeld (Pool T)	SSI Diagnostica	#16743
ImmuLex^{TM} Pneumococcus Antisera (Pool A)	SSI Diagnostica	#52390
ImmuLex^{TM} Pneumococcus Antisera (Pool B)	SSI Diagnostica	#52391
ImmuLex^{TM} Pneumococcus Antisera (Pool D)	SSI Diagnostica	#52393
ImmuLex^{TM} Pneumococcus Antisera (Pool E)	SSI Diagnostica	#52394
ImmuLex^{TM} Pneumococcus Antisera (Pool G)	SSI Diagnostica	#52396
ImmuLex^{TM} Pneumococcus Antisera (Pool H)	SSI Diagnostica	#52397
ImmuLex^{TM} Pneumococcus Antisera (Pool P)	SSI Diagnostica	#52399
ImmuLex^{TM} Pneumococcus Antisera (Pool Q)	SSI Diagnostica	#52400
ImmuLex^{TM} Pneumococcus Antisera (Pool R)	SSI Diagnostica	#52401
ImmuLex^{TM} Pneumococcus Antisera (Pool S)	SSI Diagnostica	#52402
ImmuLex^{TM} Pneumococcus Antisera (Pool T)	SSI Diagnostica	#52403
mAb Culture Supernatant serotype 11A	SunFire Bio	#TCS-11AM02
mAb Culture Supernatant serotype 15B	SunFire Bio	#TCS-15BG05
mAb Culture Supernatant serotype 19A	SunFire Bio	#TCS-19AG01
mAb Culture Supernatant serotype 23F	SunFire Bio	#TCS-23FG03
Goat Anti-Rabbit IgG Micro-Beads	Miltenyi Biotec	#130-048-602: AB_244362
Rat Anti-mouse IgM Micro-Beads	Miltenyi Biotec	#130-047-301: AB_244358
Goat Anti-mouse IgG Micro-Beads	Miltenyi Biotec	#130-048-402: AB_244361
**Bacterial and virus strains**		
S. pneumoniae serotype 12F	CDC Collection	#ABC010018244
S. pneumoniae serotype 23F	CDC Collection	#ABC020007103
S. pneumoniae serotype 3	CDC Collection	#ABC020026160
S. pneumoniae serotype 14	Clinical Isolate (Ben-Gurion University, Israel)	#107 (W4951)
S. pneumoniae serotype 19A	Clinical Isolate (Ben-Gurion University, Israel)	#109 (W5120)
S. pneumoniae serotype 15A	CDC Collection	#ABC020030426
S. pneumoniae serotype 11A	CDC Collection	#ABC020009080
S. pneumoniae serotype 35B	Clinical Isolate[30]	Pool_hung_B5 (H_22)
S. pneumoniae serotype D39	Jason Roche's Laboratory Stock	N/A
S. pneumoniae D39Δcps::SweetJanus	Echlin et al.,[11]	N/A
**Biological samples**		
Pneumococcal-negative Saliva	This study	N/A
Pneumococcal-positive Saliva Samples	Wyllie et al.,[21]	A720, C677

(*Continued on next page*)

Continued		
REAGENT or RESOURCE	SOURCE	IDENTIFIER
Pneumococcal-positive Saliva Sample	Wyllie et al.,[23]	41.2
Pneumococcal-positive Saliva Samples	Rayack et al.,[22]	#57, #120, #125,
Chemicals, peptides, and recombinant proteins		
CSP-1	ANASPEC	#AS-63779
CSP-2	ANASPEC	#AS-63877
Sheep Blood Defibrinated	Colorado Serum Company	#31125
Luna® Universal Probe qPCR Master Mix	New England Biolabs	#M3004
iQ SYBR Green Supermix	BioRad	#1708882
BSA	Americanbio	#AB01088-00100
Penicillin G sodium salt	Sigma	#P3032
Streptomycin sulfate salt	SIGMA S9137	#S9137
Kanamycin Sulfate	Americanbio	#AB01100-00010
1xPBS	Crystalgen	#221-132-05
Glycerol	Sigma	#G7893
Gentamycin Reagent Solution	Gibco	#15710–064
Diagnostic Disks (optochin)	Oxoid	#DD0001B
Bacto™ Todd Hewitt Broth	BD Biosciences	#249240
BBL™ Trypticase™ Soy Agar Modified	BD Biosciences	#212305
Bacto™Yeast Extract, Technical	Gibco	#288620
Sucrose, Crystal	J.T.Baker	#4072–01
Ethyl alcohol, Pure	Sigma	#E7023
Chloroform-isoamyl alcohol mixture	Sigma	#25666
Phenol-chloroform-isoamyl alcohol mixture	Sigma	#77617
Critical commercial assays		
MagMAX™ Viral/Pathogen Binding Solution	Applied Biosystems by Thermo Fisher Scientific	#A42359
MagMAX™ DNA/RNA Binding Beads	Applied Biosystems by Thermo Fisher Scientific	#A42362
MagMAX™ Viral/Pathogen Wash Solution	Applied Biosystems by Thermo Fisher Scientific	#A42360
MagMAX™ Elution Solution	Applied Biosystems by Thermo Fisher Scientific	#A42364
MagMAX™ Proteinase K	Applied Biosystems by Thermo Fisher Scientific	#A42363
Oligonucleotides		
PiaB Forward (CATTGGTGGCTTAGTAAGTGCAA)	Eurofins USA	N/A
PiaB Reverse (TACTAACACAAGTTCCTGATAAGGCAAGT)	Eurofins USA	N/A
PiaB Probe (TGTAAGCGGAAAAGCAGGCCTTACCC)	Eurofins USA	N/A
Other		
Phase Lock Gel Light	Quantabio	#2302820
Pharma KingFisher™ Flex 96 Deep-Well Plates	Applied Biosystems by Thermo Fisher Scientific	#A43075
Pharma KingFisher™ Flex 96 Standard Plates	Applied Biosystems by Thermo Fisher Scientific	#A43076
Pharma KingFisher™ Flex 96 Deep-Well Tip Combs	Applied Biosystems by Thermo Fisher Scientific	#A43074

RESOURCE AVAILABILITY

Lead contact

Further information and requests for resources and reagents should be directed to and will be fulfilled by the lead contact, Anna York (anna.york@yale.edu).

Materials availability

This study did not generate new unique reagents.

Data and code availability

- All data reported in this paper will be shared by the lead contact upon request.
- This paper does not report original code.
- Any additional information required to reanalyze the data reported in this paper is available from the lead contact upon request.

EXPERIMENTAL MODEL AND SUBJECT DETAILS

Microbe strains

Invasive pneumococcal disease isolates were obtained from the Centers for Disease Control/Active Bacterial Core surveillance isolate bank, and carriage isolates were obtained from our isolate bank, comprising samples from Ron Dagan (Ben-Gurion University, Israel) and Adrienn Tothpal and Eszter Kovacs (Semmelweis University, Hungary).[30] All IPD and carriage isolates were cultured by plating onto blood agar plates (BAP) comprised of Tryptic Soy Agar (TSA) II supplemented with 5% (v/v) defibrinated sheep blood, and incubated overnight at 37°C, 5% CO_2. A lawn from a BAP was resuspended into 1mL BHI using a cotton swab and 400 μL of this was used to inoculate 40 mL BHI. Samples were grown at 37°C, 5% CO_2 and optical density (OD) at 620 nm was monitored regularly. Cells were harvested in mid-log phase (0.4–0.6 AU) by centrifugation at 3220 ×g for 10 min, and the pellet was resuspended in 10 mL BHI +10% (v/v) glycerol and stored at −80°C. D39S and D39SΔ*cps*:SweetJanus were provided by Jason Roche's lab.[11]

Human subjects

De-identified pneumococcus-negative saliva samples were obtained from healthy volunteers (<30 years of age; IRB protocol number 2000029374). Raw, untreated saliva was stored at −20°C until needed. De-identified clinical saliva samples collected from individuals enrolled and sampled in accordance with Yale University HIC-approved protocols #2000027690,[31] #2000028639[32] and #2000026100[33] were used to validate the MBS method. All study participants acknowledged that they had understood the study protocol and provided verbal- or written-informed consent. Saliva samples were processed by plating 100 μL onto BAP supplemented with 10 μg/mL gentamycin and incubated overnight. The lawn of the culture-enriched saliva was harvested into 2100 μL BHI +10% (v/v) glycerol and stored at −80°C.

METHOD DETAILS

Figure 1 summarizes the MBS method; briefly, a mixture of serotypes is incubated with antisera pool(s) unique to the desired serotype, then, following wash steps is incubated with secondary antibody conjugated to a magnetic bead. The cells are extracted using the automated Kingfisher Flex Purification System and the eluate plated on blood agar plates. Unless otherwise stated a blood agar plate (BAP) comprises Tryptic Soy Agar (TSA) II supplemented with 5% (v/v) defibrinated sheep blood, and are sometimes referred to as 'plain plates'. BAPs containing the following concentrations of antibiotics/additives for selection were also used: 0.018 μg/mL, 0.036 μg/mL, 0.18 μg/mL and 0.072 μg/mL penicillin, 10 μg/mL gentamycin, 400 μg/mL kanamycin and 800 μg/mL streptomycin with 10% (w/v) sucrose. Unless otherwise stated all overnight incubations occur at 37°C and 5% CO_2.

Magnetic bead-based separation (MBS) method

Approximately 1×10^4 cells and 1×10^7 cells from two different serogroups of *S. pneumoniae* were mixed together (~0.1% minority serotype). Cells were pelleted by centrifugation at 18,516 ×g and resuspended in 450 μL Buffer 1 (1x PBS with 1% BSA). The resuspended sample was incubated at 4°C on a shaking platform at 150 rpm for 1 h. The two antisera pools specific for the minority serogroup were combined in a 1:1 ratio and diluted 50-fold in Buffer 1. Next, 30 μL of antisera mix was added to the sample and incubated at 4°C on a shaking platform at 150 rpm for 1 h. The sample was centrifuged at 18,516 ×g for 5 min, the supernatant was discarded, and the pellet was resuspended in 450 μL Buffer 1; this step was repeated again. Next, 20 μL of Anti-Rabbit IgG Micro-Beads (Miltenyi Biotech) was added, gently vortexed and incubated at 4°C on a shaking platform at 150 rpm for 30 min. The sample was extracted using the KingFisher™ Flex Purification System (ThermoFisher) with the protocol detailed in Table S1. The eluted sample was resuspended by pipetting the sample in the elution well 50–100 times before transferring it to a new Eppendorf tube. Following transfer, the sample was thoroughly mixed by vertexing a minimum of 10 times for 5–10s with 5 s intervals.

To minimize cell losses, when supernatant was removed from cell pellets, 50 μL of supernatant was always left on top of the pellet. The specific rabbit antiserum pools (SSI Diagnostica, Hillerød, Denmark) used for the MBS method, and the SSI ImmuLex™ Pneumotest Pools used for serotyping are outlined in Table S2.

Proof of concept and primary analysis

To demonstrate proof of concept for the MBS method we used three pairs of six different serotypes where one serotype in each pair was penicillin resistant and the other penicillin sensitive. It is important to note that different penicillin sensitivity is not necessary for separation but was instead used to make the quantification of the efficiency of this method easier. The three pairs were 12F and 23F (Pair 1), 3 and 14 (Pair 2) and 19A and 15A (Pair 3). Serotype 3 exists as two distinct morphologies; small non-mucoid colony variant (SCV) and mucoid variant.[34] We therefore isolated SCV and mucoid variants and chose to work primarily with the SCV for three

reasons; SCVs are easier to count, easier to isolate as single colonies (for serotyping) and less easy to distinguish from other serotypes based on morphology, thus reducing selection bias during the colony selection for serotyping. The MIC of each serotype was determined using penicillin E-strips, and then the exact concentration of penicillin for blood agar plates was determined experimentally by varying the penicillin concentration and plating out cells at known CFU/mL. The concentration of penicillin used in the blood agar plates was the concentration at which the resistant serotype grew equally well on a penicillin containing plate, as it did on a plain plate, whilst the susceptible serotype showed no growth on the penicillin containing plate but normal growth on a plain plate. For Pairs 1, 2 and 3, BAPs containing 0.018 μg/mL, 0.036 μg/mL and 0.18 μg/mL penicillin were used, respectively.

For all three pairs, Sample R is when the penicillin resistant serotype is the minority species, and Sample S is when the penicillin sensitive serotype is the minority species. Samples were plated out onto BAPs with and without penicillin, at two stages in the protocol; immediately prior to the first incubation (PRE), and after extraction (POST). In all cases 5 μL of sample was serially diluted in 45 μL PBS, in triplicate. For samples where the minority strain was penicillin resistant, 20 μL of sample at a 10^{-1} dilution was plated on penicillin plates, while 20 μL of sample at a 10^{-4} dilution was plated on plain blood agar plates. In samples where the majority serotype was penicillin resistant, 20 μL of sample at a 10^{-4} dilution was plated on both BAPs with and without penicillin. In addition to the diluted samples, 10 μL of undiluted sample at the PRE and POST stage, and the remaining volume (~40 μL) after elution was plated on BAPs, to provide DNA for qPCR experiments conducted to establish separation efficiency. In all cases 10 μL or 20 μL samples were pipetted onto the BAP and the plate was then tilted to allow the sample to run down the length of the plate. The BAPs were incubated overnight.

Secondary analyses

To establish if separation efficiency was similar for both mucoid (Muc) and small colony variants (SCV) of Serotype 3, two additional pairs; 23F and 3SCV (Pair 4), and 23F and 3Muc (Pair 5) were investigated. These experiments were conducted in duplicate, and efficiency assessed by colony counting and qPCR methods. Pair 4 and 5 used BAPs containing 0.072 μg/mL penicillin.

To investigate the effect of initial proportion of minority serotype on the efficiency of separation, 23F and 12F (Pair 1) were again used. The initial amount of majority serotype (12F) was kept constant at 1×10^7 CFU, while the minority serotype (23F) was varied (5×10^4, 1×10^4, 5×10^3 and 1×10^3). These experiments were conducted once for each dilution, and efficiency was assessed by colony counting and qPCR methods.

The experiments above were conducted using two pooled antisera that were specific for the minority serotype. We investigated whether a single pool of antisera could also be used. This is important because certain pairs of serotypes can only be distinguished by one pool. Serotype pairs which could not be distinguished based on penicillin sensitivity (and therefore could not be assessed by colony counting methods), were used for this analysis, and for pairs which shared a common antisera pool, only the unique antisera was used. These experiments were conducted once for each condition, and efficiency was assessed by qPCR alone.

Colony counting to quantify separation efficiency

Colonies were counted and the mean colony number was determined, which was then used for downstream analysis. The following equations for Sample R and Sample S were used to determine the percentage of the minority serotype present at each time point.

Sample R equation

$$\%\ minority = \left(\frac{\#colonies\ (pen\ plate)}{\#colonies\ (plain\ plate)}\right) \times 100$$

Sample S equation

$$\%\ minority = \left(\frac{\#colonies(plain\ plate) - \#colonies(pen\ plate)}{\#colonies(plain\ plate)}\right) \times 100$$

Serotyping of colonies to confirm separation efficiency

Eight colonies were picked at random from the plain blood agar elution plates and expanded to create a lawn on $1/8^{th}$ of a BAP and incubated overnight. The serotype of each lawn was confirmed by testing each of the four antisera pools specific to both the majority and the minority serotype in the pair, using ImmuLex™ Pneumotest (SSI Diagnostica) reagents.

Real-time qPCR to confirm separation efficiency

Colonies/lawns from each sample, grown on BAP, were harvested into 200 μL PBS using a cotton swab and the DNA was extracted using a DNeasy Blood and Tissue Kit (QIAGEN) as per the manufacturers protocol. DNA concentration was measured using Qubit™ as per the manufacturers protocol. A no-template negative control was included for each primer pair used,[35] and a standard curve (positive control) was constructed using genomic DNA from each of the six serotypes under investigation. Each qPCR reaction was

25 μL total volume, consisting of iQ™ SYBR® Green Supermix (BioRad), 5 μL of template DNA and 200 nM of each primer. The real time qPCR was run on a BioRad CFX96™ Touch Real-Time qPCR System. The cycling conditions were 1 cycle of denaturation at 95°C for 10 min, followed by 40 cycles of 95°C for 15 s and 60°C for 1 min for amplification, and a melt curve from 65°C to 95°C in increments of 0.5°C. For each sample, amplification with primer pairs from both the minority and majority serotype was conducted in duplicate, the mean of duplicates was used for downstream analysis. The concentration of each serotype in a sample was determined by comparing the C_T value to the standard curve for the corresponding serotype.

Demonstrating applications for the MBS method

Generation of capsule-switch mutants by transformation

To determine whether the MBS method could be used to improve capsule switching experiments (by reducing workload and scaling-up transformations), genomic DNA (gDNA) from four donor serotypes (12F, 23F, 35B, 11B) was transformed individually and as a mixed sample into the recipient D39SΔ*cps*:SweetJanus.[11] The mixed sample was processed with and without the use of the MBS method. An individual transformation of D39 gDNA into D39SΔ*cps*:SweetJanus was included as a positive control.

With the exception of using Todd Hewitt supplemented with 0.5% Yeast Extract (THY) media for liquid cultures, gDNA was extracted as outlined previously.[11] Briefly, the pneumococcal isolate was grown as a lawn on BAP overnight at 37°C and 5% CO_2. The lawn was harvested into 1 mL BHI and centrifuged at 18,516 ×g, the cell pellet was resuspended in 1 mL resuspension buffer (25 mM Tris-HCl, pH 8.5, 10 mM EDTA, pH 8.5, 25 mM Glucose, 250 mg RNase) supplemented with 50 μL 10% SDS50 μL 10% Deoxycholate and 10 μL proteinase K. Sample was incubated for 5–10 min at 37°C and ⅓ volume added to 3 phase-lock tubes, 500 μL Phenol:Chloroform:Isoamylalcohol was added to each tube and samples were inverted ten times. Samples were centrifuged for 5 min at 18,516 ×g, 500 μL Chloroform:Isoamylalcohol was added to each tube and samples were inverted ten times. Samples were centrifuged for 5 min at 18,516 ×g and aqueous layers were combined and added to 7 mL ice-cold 100% Molecular Grade Ethanol. Precipitated DNA was pelleted by centrifugation at 3220 ×g. Pellet was washed twice in 70% ethanol and dried before resuspension in 200 μL nuclease-free water.

Frozen stocks of D39SΔ*cps*:SweetJanus were inoculated onto BAP and incubated overnight. Cells harvested from the BAP were used to inoculate Todd Hewitt supplemented with 0.5% Yeast Extract (THY) media to a starting OD_{620} of 0.04 AU, and were grown at 37°C and 5% CO_2 until OD_{620} =∼0.08). For each of the five individual transformations, 1 mL of culture was transferred into a 1.5mL Eppendorf tube, 3 μg/mL of competence stimulating peptide 1 (CSP1) and 4 μg of the appropriate DNA was added. For the mixed transformation, 4 mL of culture was transferred to a 15 mL falcon tube, 3 μg/mL CSP1 and 4 μg of each of the four gDNA templates was added. Cells were incubated for 3 h at 37°C. Subsequently, individual transformation and mixed transformation samples were positively selected for by plating on BAP supplemented with 800 μg/mL streptomycin and 10% (w/v) sucrose (Strep/Suc plates), and incubated overnight.

For the five samples that underwent individual transformations, eight colonies each were selected and expanded onto new Strep/Suc plates and incubated overnight. These expanded samples were re-plated onto both Strep/Suc plates, as well as BAP supplemented with 400 μg/mL kanamycin (Kan plates), for negative selection, and incubated overnight. Colonies that grew on Strep/Suc but not Kan plates were serotyped to confirm they have successfully gained the capsule.

For the mixed transformation sample, all colonies were harvested using a cotton swab and resuspended in 1.5 mL Brain Heart Infusion (BHI) media +10% (v/v) glycerol. As a control, 100 μL of the mixed sample was serially diluted to 10^{-6}, then 100 μL of 10^{-4}, 10^{-5} and 10^{-6} dilutions were plated on BAP, and incubated overnight. Following, 100 μL of the mixed sample was aliquoted into four 1.5 mL Eppendorf tubes, centrifuged at 18,516 ×g resuspended in 500 μL Buffer 1 and processed through MBS using the appropriate antisera pool(s) for targeting the appropriate serotype. The elution was plated on BAP and incubated overnight. Thirty-two colonies were selected from the mixed sample that did not undergo MBS, and eight colonies were selected from each of the four samples that had undergone MBS. The serotype of all expanded colonies was determined using SSI latex agglutination.

Isolating pneumococci from a saliva sample

The relationship between qPCR cycle threshold (C_T) value and CFU/mL was determined using pneumococcus-negative saliva, spiked with pneumococci (serotype 19A) at a variety of known CFU/mL. The concentration of the 19A stock was determined to be 5×10^9 CFU/mL, which was then serially diluted 1:10 in pneumococcus-negative saliva. After 2 h at room temperature, 100 μL of each sample was plated onto BAP supplemented with 10 μg/mL gentamycin (Gent plates) and incubated overnight. The lawn of each culture-enriched saliva sample was harvested into 2100 μL BHI +10% (v/v) glycerol using an L-shaped spreader. DNA was extracted using the MagMAX Viral/Pathogen Nucleic Acid Isolation Kit with a modified protocol,[33] briefly, 200 μL sample and 10 μL proteinase K were added to a single well of a deep-well block; 1mL of Wash Buffer, 1mL of 80% ethanol and 0.5mL ethanol were added to the corresponding wells on three other deep-well blocks (Wash1, 2 and 3 respectively) and 90 μL of elution solution was added to corresponding wells on two elution plates. Plates were loaded into the Kingfisher Apex (ThermoFisher Scientific) and a standard protocol (Table S3) was executed. When prompted by the machine, 10 μL proteinase K, 530 μL Binding solution and 25 μL Magnetic Beads were added to the sample well, the extraction protocol was resumed. All DNA templates were tested by qPCR for the pneumococcal gene *piaB*[36,19] using Luna® Universal One-Step RT-qPCR mix, 2.5 μL template DNA and 200 nM of each primer and probe in a total reaction volume of 20 μL. The cycling conditions were 1 cycle of denaturation at 95°C for 3 min, followed by 40 cycles of 98°C for 15 s and 60°C for 30 s. C_T values were plotted against CFU/mL of 19A in the raw saliva sample (Figure S1).

Using data from Figure S1 in combination with data from previous studies[28] we were able to determine suitable concentrations for spiked-saliva, that reflect levels commonly found in saliva obtained from the healthy individuals during carriage studies. Pneumococcus-negative saliva was spiked with pneumococci (serotype 19A) at varying concentrations (5×10^4, 5×10^3, 5×10^2 and 5×10^1 CFU/mL) and left at room temperature for 2 h. Following, 100 μL of each sample was plated onto Gent plates and incubated overnight. The lawn of the culture-enriched saliva was harvested into 2100 μL BHI +10% (v/v) glycerol.

From each culture-enriched saliva sample, 10 μL was added to 490 μL Buffer 1, and cell separated using the MBS protocol, with the following modifications. The primary incubation step was conducted using 15 μL SSI antisera (1:50 dilution) and 5 μL of the appropriate SunFire Bio monoclonal antibody (mAb) combined. Where the use of SSI antisera and SunFire Bio mAb was not successful, SSI antisera alone was tested. The secondary incubation was conducted using 15 μL of anti-mouse IgG or IgM Micro-Beads (Miltenyi Biotech), as appropriate, to target the mAb only. For samples using only SSI antisera, 15 μL of anti-rabbit IgG Micro-Beads (Miltenyi Biotech) were used in the secondary incubation. As a negative control, culture-enriched saliva samples were also processed in the absence of MBS, these samples were serially diluted in PBS to 10^{-6}, the 10^{-4}, 10^{-5} and 10^{-6} dilutions were plated on BAPs and incubated overnight.

Colonies that looked like pneumococci (small, gray, moist colonies with a green zone of alpha-hemolysis), were isolated and expanded onto new BAP. Each expanded colony was optochin tested to confirm whether it was pneumococcus (optochin sensitive) or another oral bacteria (optochin resistant). Where a ring of optochin sensitivity was observed but a second (contaminating) bacteria with optochin resistance was also present or, where satellite colonies of pneumococcus were present within the zone of inhibition, samples were considered 'pneumococcal colonies' since pure pneumococci can be isolated from the contaminant.

QUANTIFICATION AND STATISTICAL ANALYSIS

Where samples were conducted in biological triplicate, average (mean) data was presented (Figure 2).

Cell Reports Methods, Volume *3*

Supplemental information

Magnetic bead-based separation

# of pneumococcal serotypes

Anna York, Emily Huynh, Sidiya Mbodj, Devyn Yolda-Carr, Maikel S. Hislop, Haley Echlin, Jason W. Rosch, Daniel M. Weinberger, and Anne L. Wyllie

Supplemental Information

Table S1. KingFisher Flex MBS Protocol. Related to STAR Methods.

**Pick up tips**
96DW tip comb
**Collect Beads**
Count 2 seconds 5
**Mix**
Release Beads<br>Medium mix 0:01:00min<br>Count 3 seconds 1
**Release Beads**
Fast 00:00:05
**Leave**
Leave Plate

Table S2. Serotypes and corresponding antisera pools used for MBS (rabbit antiserum; SSI Diagnostica, Hillerød, Denmark) and for serotyping (ImmuLex™ Pneumotest; SSI Diagnostica).Related to STAR Methods.

**Serotype**	**Pooled Antisera for Neufeld**	**ImmuLex™ Pneumococcus Antisera**
12F	Pool E #16733<br>Pool R #16741	Pool E #52394<br>Pool R #52401
23F	Pool H #16736<br>Pool Q #16740	Pool H #52397<br>Pool Q #52400
3	Pool B #16728<br>Pool R #16741	Pool B #52391<br>Pool R #52401
14	Pool P #16739<br>Pool H #16736	Pool P #52399<br>Pool H #52397
19A	Pool B #16728<br>Pool P #16739	Pool B #52391<br>Pool P #52399
15A/B/C	Pool H #16736<br>Pool S #16742	Pool H #52397<br>Pool S #52402
2 (D39)	Pool A #16725<br>Pool T #16743	Pool A #52390<br>Pool T #52403
35B	Pool G #16735	Pool G #52396
11A/B	Pool D #16731<br>Pool T #16743	Pool D #52393<br>Pool T #52403

Table S3. KingFisher Flex DNA extraction protocol. Related to STAR Methods.

**Pick up tips**
96DW tip comb
**Predigest**
56ºC<br>Preheat on<br>Mixing medium 0:10:00 minutes
**Heat kill pk 95 ºC**
Preheat off<br>Mixing medium 0:10:00 minutes
**Initial Mixing**
Release bead 0:00:00<br>Slow mix 0:01:00min<br>Medium mix 0:04:00min<br>Loop1
**Add Proteinase K and Binding**
Add 10 µl pro k, 25 µl binding beads and 530 µl binding buffer
**PK Digest**
Release bead 0:00:00<br>Preheat on 65ºC<br>Mix Fast 0:15:00 min<br>Collect beads 5 count 1 second<br>Collect beads: Count 5 time 0
**Wash 1**
Release beads 20 second bottom mix<br>Mixing 0:00:10 bottom mix<br>0:00:10 Fast mix<br>Loop 3 times<br>Collect beads: Count 5
**Wash 2**
Release beads<br>0:00:20 FAST<br>Mixing 0:00:10 bottom mix<br>0:00:10 Fast mix<br>Loop 2 times<br>Collect beads: 4 count 1 second
**Wash 3**
Release beads<br>0:00:00<br>Mix<br>0:00:30 Fast<br>Collect beads: Count 5 seconds 0
**Dry**
0:02:00 Dry type (outside well)
**Elution 1**
Release Beads<br>0:00:00<br>Heating and Cooling<br>Preheat ON<br>75°C<br>Mixing 0:00:15 bottom mix<br>0:00:45 Medium mix<br>Loop 6 times<br>Collect Beads<br>Count 1 seconds 0

**Final Beads**
Mixing<br>0:02:00 slow
**Elution 2**
Release Beads<br>0:00:00<br>Heating and Cooling<br>Preheat ON<br>75°C<br>Mixing 0:00:15 bottom mix<br>0:00:45 Medium mix<br>Loop 6 times<br>Collect Beads<br>Count 1 seconds 0
**Final Beads**
Mixing<br>0:02:00 slow
**Leave Tip**
Tip Plate
**HOLD Temp**
Elution Plate 1<br>10°C

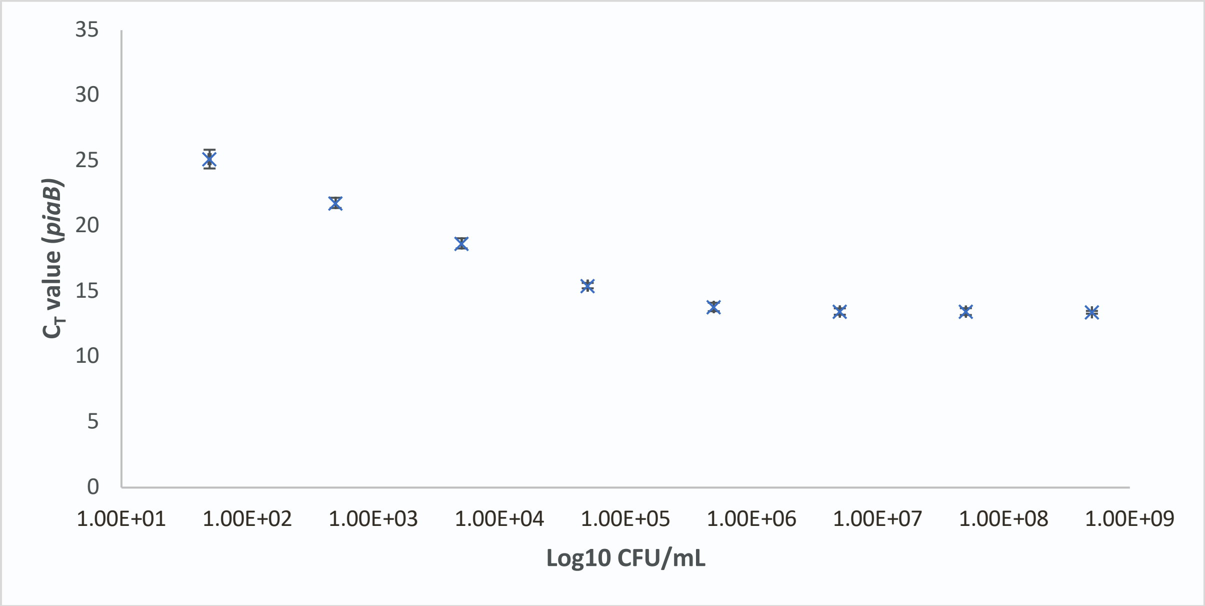

Figure S1. Detection of pneumococcus gene piaB (C_T value) when culture-enriched saliva samples were tested with qPCR, and the corresponding CFU/mL of S. pneumoniae 19A that was spiked into each raw saliva sample. Raw saliva was confirmed to be pneumococcus-negative (C_T >40) by qPCR towards piaB. Data shown as mean and standard deviation of biological triplicate data. Related to STAR Methods.
